# Intragenic antimicrobial peptides (IAPs) from human proteins with potent antimicrobial and anti-inflammatory activity

**DOI:** 10.1371/journal.pone.0220656

**Published:** 2019-08-06

**Authors:** Guilherme D. Brand, Marcelo H. S. Ramada, Júlia R. Manickchand, Rafael Correa, Dalila J. S. Ribeiro, Michele A. Santos, Andreanne G. Vasconcelos, Fernando Y. Abrão, Maura V. Prates, André M. Murad, José L. Cardozo Fh, José Roberto S. A. Leite, Kelly G. Magalhães, Aline L. Oliveira, Carlos Bloch

**Affiliations:** 1 Laboratório de Síntese e Análise de Biomoléculas, LSAB, Instituto de Química, Universidade de Brasília, Brasília, DF, Brasil; 2 Programa de Pós-Graduação em Ciências Genômicas e Biotecnologia, Universidade Católica de Brasília, Brasília, DF, Brasil; 3 Programa de Pós-Graduação em Gerontologia, Universidade Católica de Brasília, Brasília, DF, Brasil; 4 Laboratório de Espectrometria de Massa, LEM, Embrapa Recursos Genéticos e Biotecnologia, Brasília, DF, Brasil; 5 Laboratório de Imunologia e Inflamação, LIMI, Instituto de Biologia, Universidade de Brasília, Brasília, DF, Brasil; 6 Laboratório de Ressonância Magnética Nuclear, LRMN, Instituto de Química, Universidade de Brasília, Brasília, DF, Brasil; 7 Núcleo de Pesquisa em Morfologia e Imunologia Aplicada, NuPMIA, Faculdade de Medicina, Campus Universitário Darcy Ribeiro, Universidade de Brasília, Brasília, DF, Brasil; 8 Faculdade de Farmácia, FacUnicamps, Goiânia, GO, Brasil; 9 Departamento de Fitopatologia, Instituto Mato-Grossense do Algodão, Primavera do Leste, MT, Brasil; Universidade Guarulhos, BRAZIL

## Abstract

Following the treads of our previous works on the unveiling of bioactive peptides encrypted in plant proteins from diverse species, the present manuscript reports the occurrence of four proof-of-concept intragenic antimicrobial peptides in human proteins, named Hs IAPs. These IAPs were prospected using the software Kamal, synthesized by solid phase chemistry, and had their interactions with model phospholipid vesicles investigated by differential scanning calorimetry and circular dichroism. Their antimicrobial activity against bacteria, yeasts and filamentous fungi was determined, along with their cytotoxicity towards erythrocytes. Our data demonstrates that Hs IAPs are capable to bind model membranes while attaining α-helical structure, and to inhibit the growth of microorganisms at concentrations as low as 1μM. Hs02, a novel sixteen residue long internal peptide (KWAVRIIRKFIKGFIS-NH_2_) derived from the unconventional myosin 1h protein, was further investigated in its capacity to inhibit lipopolysaccharide-induced release of TNF-α in murine macrophages. Hs02 presented potent anti-inflammatory activity, inhibiting the release of TNF-α in LPS-primed cells at the lowest assayed concentration, 0.1 μM. A three-dimensional solution structure of Hs02 bound to DPC micelles was determined by Nuclear Magnetic Resonance. Our work exemplifies how the human genome can be mined for molecules with biotechnological potential in human health and demonstrates that IAPs are actual alternatives to antimicrobial peptides as pharmaceutical agents or in their many other putative applications.

## Introduction

Sometimes, Art may offer better ways than Hard Sciences to find truth and understand reality. The Shakespearian remark "Let every eye negotiate for itself and trust no agent” (W. Shakespeare—Much Ado About Nothing. Act 2, scene 1) is a fair example of that, if one is willing to consider some aspects of our present knowledge on protein structure, function and dynamics.

Proteins can be seen and studied under several different “eyes”. In brief, just some of them: a) simply as a linear biopolymer, product of the translation of RNA molecules by ribosomes in the cytoplasm; b) as unique sequences of covalently linked amino acid residues that serve as substrate for enzymes that catalyze various post-translation modifications; c) as a set of peptide building blocks, secondary structures, domains that are determinant to their final tridimensional folding and mature topology; d) or as molecules exposed to large—occasionally dramatic—internal motions and conformational changes induced by solvents, small ligands, proteins and other macromolecules in order to perform their biological functions under certain physicochemical conditions.

For more than a decade our research group has decided to systematically “see” proteins as sources of encrypted bioactive peptides [1]. Initially, the rationale behind this approach was inspired by the fact that several peptide sequences were identified and reported as free fragments under specific physiologic conditions, structurally independent and autonomous bioactive entities derived from their native parent-proteins. Caseins, hemoglobins, interleukins, among others, are known to host a variety of these fragments showing in vivo biological functions markedly different from their much larger, intact, mature polypeptide chain donors [2–5]. In the course of time, our work progressively demonstrated that this phenomenon could be observed in a wider range of proteins with little or no significant evolutionary relation to the previous ones [6–8]. Moreover, similarly to some protein folding methodological studies that “sees” the polypeptide chain as an assembly of particular sub-sets of smaller fragments orderable in a hierarchical process to yield the final protein architecture, such as: building blocks (microdomains), hydrophobic folding units (subdomains), protein domain, protein fold (multidomains) and quaternary structure [9–14], we have also located inside a number of protein primary structures, shorter amino acid sequences active against pathogenic microorganisms, potent opioids and hypotensive agents [6,7].

The present work represents an additional step towards a broader evaluation of our prime hypothesis by searching for different biological functions—yet to be revealed using the present approach—and, at the same time to challenge our system with different examples that could reinforce and/or improve our leading searching tool, an *in-house* developed software named Kamal. Kamal was used in proof-of-concept publications [6,7] and was recently submitted for publication and software distribution to the scientific community. In this opportunity, we report the uncovering as well as the biochemical and biophysical evaluations of four new representative peptides derived from the internal sequences of human proteins, called Hs IAPs, or *Homo sapiens* Intragenic Antimicrobial Peptides. Human cells express a variety of antimicrobial peptides (AMPs) to hinder the proliferation of opportunistic bacteria, yeasts and viruses [15]. These so-called Host Defense Peptides (HDPs) have direct antimicrobial activity and orchestrate, in a complex and often uncontrolled manner, the immune system in case of injury [16–20]. Some of these molecules, such as the cathelicidin LL-37, have also been tested as antimicrobials for topical application in polymicrobial infected wounds [21,22]. It is our prime hypothesis that human proteins are source material for encrypted peptides with potent and broad antimicrobial activity as well as immunomodulatory potential, and therefore can be considered alternatives to HDPs in various applications, such as topical pharmaceutical agents.

Unlike our previous works, here we demonstrate that the novel Hs IAPs not only disturb the main phase transition of model membranes, fold into amphiphilic α-helical segments upon binding to model membranes and display distinct antimicrobial activities against Gram-positive, and -negative bacteria as well as yeasts and filamentous fungi, but also one of these IAPs—Hs02 (KWAVRIIRKFIKGFIS-NH2)—derived from the unconventional myosin 1h protein (NP_001094891.3), is a potential inhibitor of lipopolysaccharide-induced release of pro-inflammatory mediators in murine macrophages. In addition to that, secondary structure predictions by circular dichroism experiments and a complete ^1^H NMR structure of Hs02 bound to phospholipid micelles are presented.

Lastly, fine-tuned with the opening Shakespearian quote, we hope that to the reader’s analytical “eye”, our results alone may stand up above other aspects as the most trustful part of this manuscript.

## Results

### Filtering the human genome for encrypted IAPs

**Fig 1:**
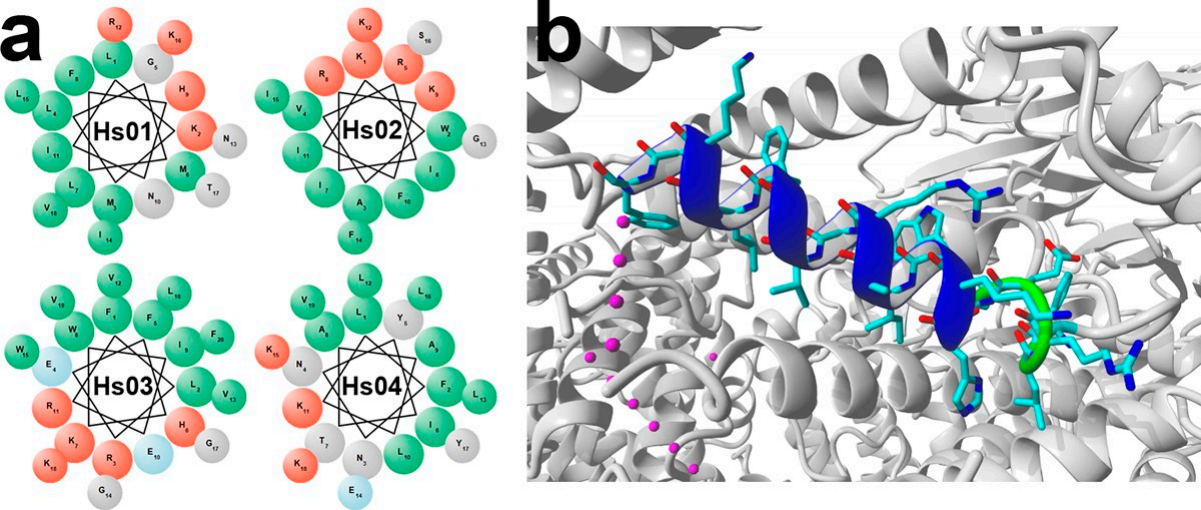
**a.** Helical wheel Edmundson plot of the peptides Hs01, Hs02, Hs03 and Hs04 highlighting their amphiphilic character. In green are hydrophobic amino acid residues, in blue, polar uncharged residues, in dark blue, negatively charged residues, and in red are positively charged residues, and **b.** Structure of the N subunit of the human Anaphase-Promoting Complex (PDB:4UI9). In detail is the α-helical segment spanning the amino acid residues from 285 to 304, which correspond to the IAP Hs03.

Nearly 160.000 *Homo sapiens* proteins were downloaded in .FASTA format from the UniprotKB website and submitted to the software Kamal v2.0. Based in the Kamal filtering procedures described in the material and methods section, 1383, 312 and 48 unique putative IAPs were found for the polar angles of θ = 192°, 160° and 128°, which derived from 349, 92, and 36 proteins, respectively. Four putative IAPs were selected for solid phase peptide synthesis to investigate whether encrypted antimicrobial peptides can be identified in human proteins using our prospective methodology. These peptides, named Hs01, 02, 03 and 04, were synthesized by solid phase peptide synthesis, purified by RP-HPLC, and verified by mass spectrometry, as described in the material and methods section. Their primary structures and source proteins are listed in Table 1. A helical wheel plot of Hs01, 02, 03 and 04 is provided to confirm that these peptides present an amphiphilic nature once structured as α-helical segments following membrane adsorption (Fig 1A). Peptide Hs03 corresponds to amino acids 285–304 of the N subunit of the human Anaphase-Promoting Complex (PDB:4UI9), and this segment is almost fully structured as an α-helix in the source protein, as demonstrated in Fig 1B. No information regarding the three-dimensional structure of the source proteins of the remaining IAPs could be obtained.

**Table 1:** Peptide code, name, primary structure, source protein and number of amino acid residues of the Hs IAPs synthesized for the present study.

Peptide code	Peptide name	Primary structure	Source protein	N. Residues
NP_001093322.2 (185–202)	Hs01	LKMLGMLFHNIRNILKTV-NH_2_	PRAME family member 20	18
NP_001094891.3 (741–756)	Hs02	KWAVRIIRKFIKGFIS-NH_2_	unconventional myosin-Ih	16
NP_037498.1 (285–304)	Hs03	FLREFHKWIERVVGWLGKVF-NH_2_	anaphase-promoting complex subunit 2	20
NP_055421.1 (582–600)	Hs04	LFNNYITAALKLLEKLYKV-NH_2_	probable E3 ubiquitin-protein ligase HERC3 isoform 1	19

### Hs IAPs are α-helical upon membrane adsorption and promote disturbances in the main phase transition of model vesicles

**Fig 2:**
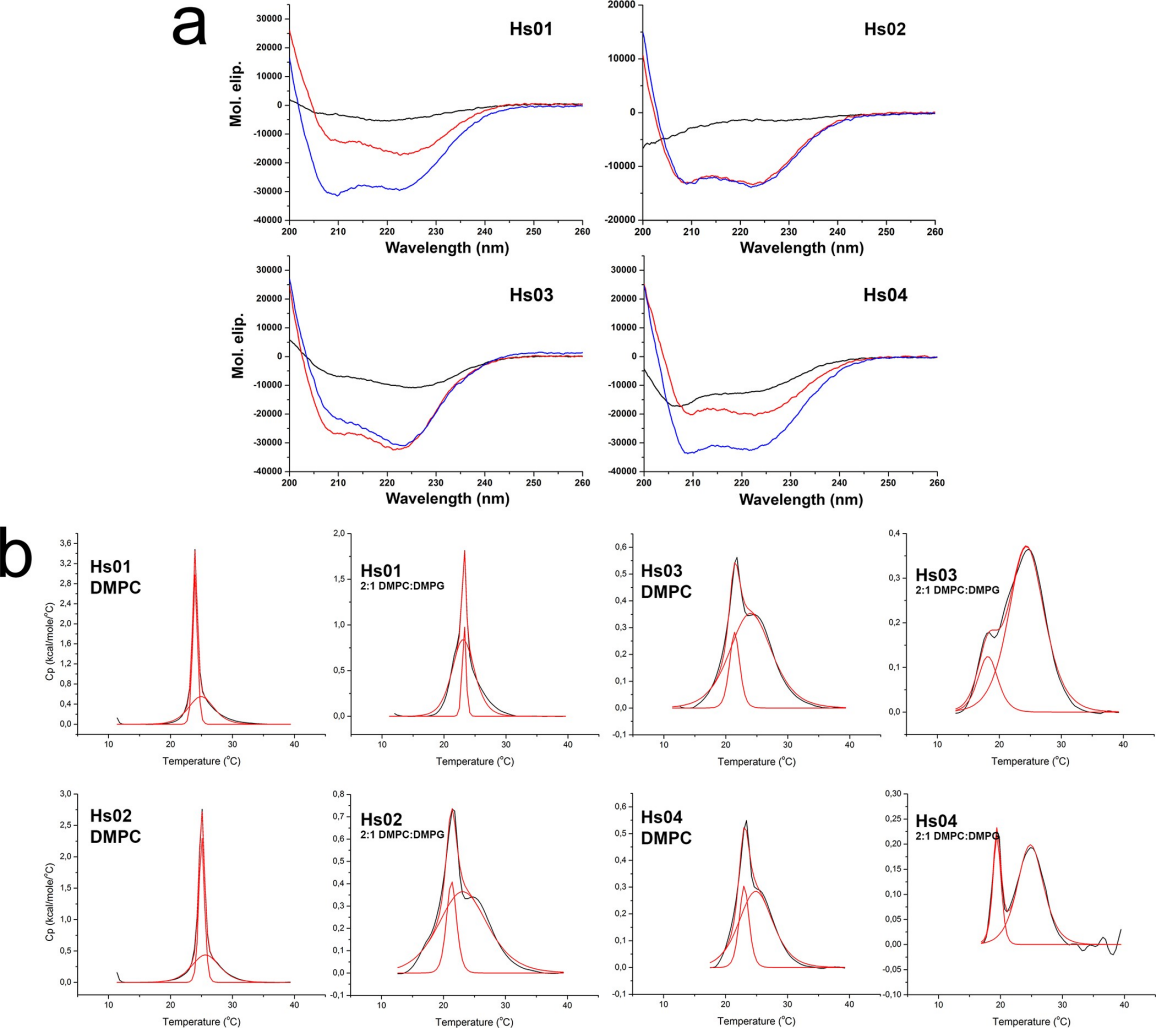
Hs IAPs transition to α-helical structure upon interaction with model phospholipid membranes and induce disturbances in the P´_β_→L_α_ phase transition of vesicles. **a.** Far-UV CD scans were performed to evaluate the secondary structure of Hs IAPs in buffer alone (40 μM solution peptide in phosphate buffer), represented as a black line, and after the addition of 2 mM DMPC, represented as a red line, or in the presence of 2 mM 2:1 DMPC:DMPG, blue line. **b.** Heating thermal scans of DMPC and 2:1 DMPC:DMPG LUVs enriched with 4 mol% IAPs. Black line corresponds to experimental data. Red line corresponds to a non-two state model fitting with two components of the main phase transition of model vesicles. Hs IAPs and the corresponding LUV compositions are indicated in each inset of the figure.

Far-UV CD scans were obtained for Hs IAPs at 40 μM concentration in phosphate buffer alone and after the addition of 50-fold molar excess of model phospholipid large unilamellar vesicles (LUVs) composed of dimyristoylphosphatidylcholine (DMPC) and 2:1 dimyristoylphosphatidylcholine: dimyristoylphosphatidylglycerol (2:1 DMPC:DMPG). Hs IAPs were mostly unstructured in buffer, although Hs03 and 04, were already nearly 30% helical in this environment (Table 2). As anticipated, the addition of phospholipid LUVs at 2 mM induced a transition to α-helical structures in the four investigated peptides, as indicated by the far-UV CD spectra (Fig 2A). The percent helicity of peptides was estimated [23] in buffer and after the addition of model phospholipid LUVs (Table 2). As previously demonstrated for other IAPs [6,7], 2:1 DMPC:DMPG LUVs induced higher percent helicity in all peptides, a common feature among AMPs and IAPs.

**Table 2:** Percent helicity of the Hs IAPs in buffer, and at a 50-fold molar excess of DMPC and 2:1 DMPC:DMPG LUVs, estimated by the method of Chen *et al*.[23].

	% Helicity Buffer	% Helicity 2:1 DMPC:PG	%Helicity DMPC
Hs01	15.1	85.9	40.3
Hs02	4.6	45.5	43.8
Hs03	29.4	88.6	92.9
Hs04	35.6	91.3	50.8

To investigate the disturbance promoted by Hs IAPs in the main phase transition of model membranes, these molecules were added at a 4 mol% ratio to DMPC and 2:1 DMPC:DMPG LUVs and then submitted to heating thermal scans in a differential scanning calorimeter (Fig 2B). DSC thermograms showed anticipated endothermic transitions characteristic of the phospholipid (P´_β_→L_α_) phase transition [6–8,24]. Thermograms were fit to a non-two state model with two components, a sharp and a broad peak, as described elsewhere [6,7]. Peptides Hs01 and Hs02 induced mild disturbances in the main phase transition of DMPC LUVs, but disturbed significantly the P´_β_→L_α_ transition of 2:1 DMPC:DMPG vesicles. Peptides Hs03 and 04 disturbed more significantly the acyl core of model membranes of both compositions. These peptides presented pronounced effects in the Transition temperature (Tm), Enthalpy (ΔH) and van´t Hoff Enthalpy (ΔH_vh_) of the broad and sharp components of LUVs.

**Fig 3:**
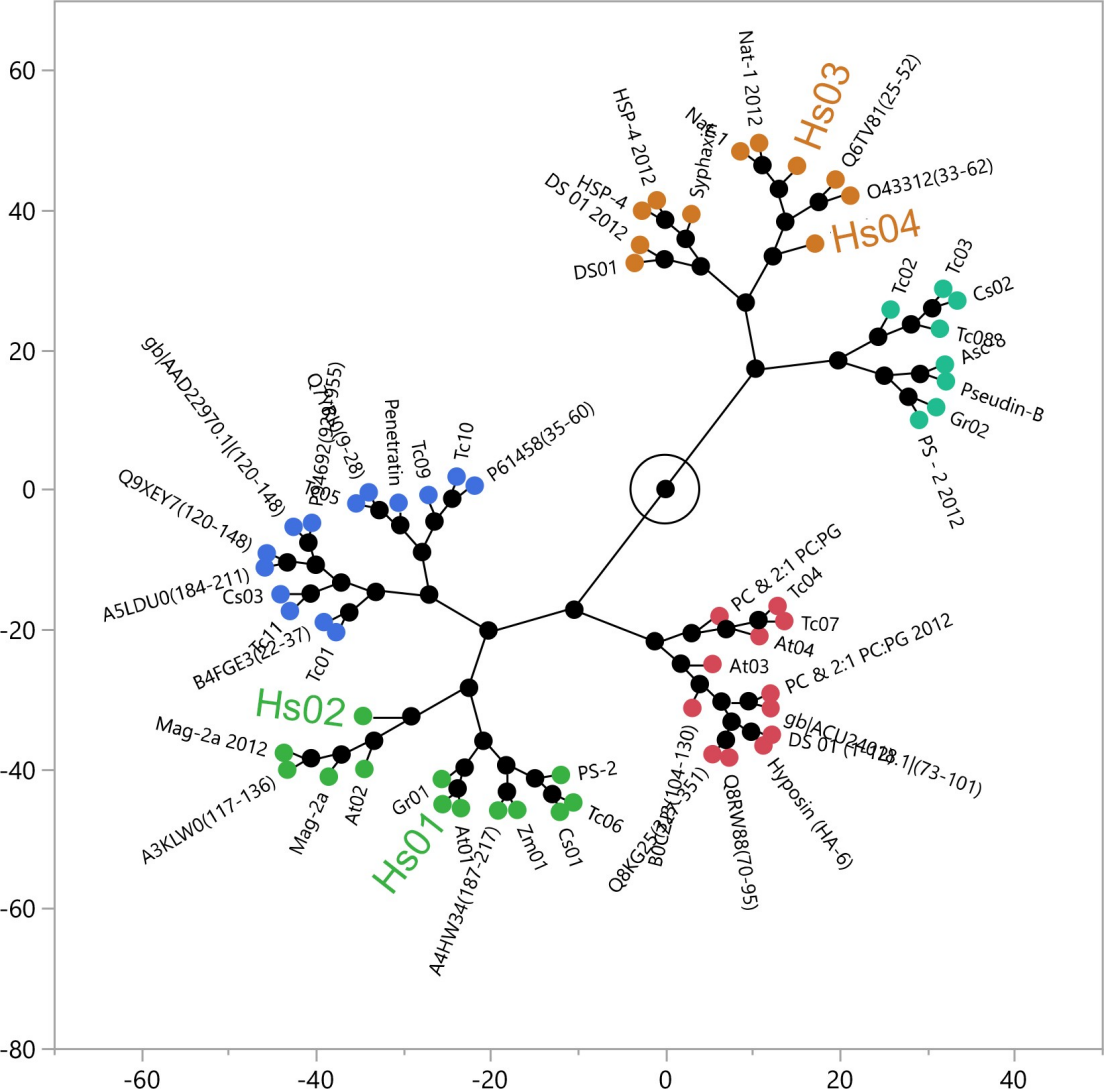
Constellation plot of the dendrogram obtained from HCA categorizes peptides in discrete clusters. The transition temperature (Tm), enthalpy (ΔH) and van’t Hoff enthalpy (ΔH_vh_) of the broad and sharp components of DMPC and 2∶1 DMPC:DMPG LUV thermograms following the addition of the Hs IAPs were obtained by adjusting peaks to a non-two state transition with two components. Data for Hs01, 02, 03 and 04 were appended to 52 other IAPs and AMPs and jointly submitted to a novel principal component analysis followed by hierarchical clustering (HCA analysis) of the first five principal components. The corresponding dendrogram is depicted in the form of a constellation plot in dimensionless units.

Individual analysis of heating thermal scans of peptide:phospholipid mixtures provides only a qualitative description of the interaction, and therefore has limited interpretation. By integrating the Hs IAPs in the framework of membrane active peptides previously introduced by our group [8], it is possible to evaluate the profile of membrane interaction these peptides display in relation to other membrane active molecules (Fig 3). The Tm, ΔH and ΔH_vh_ obtained for the sharp and broad components of the P´_β_→L_α_ transition of DMPC and 2:1 DMPC:DMPG LUVs enriched with Hs IAPs were joined to a previous set of 52 membrane active peptides and submitted to a principal component analysis. Additionally, the first five principal components were clustered using a hierarchical clustering algorithm, as previously performed by our group [8]. Such analysis indicates that Hs01 induces a thermal profile in model membranes similar to the IAPs Gr01 and At01 [7], while Hs02 induced disturbances that are comparable to the IAP At02 and the natural peptide Magainin-2a [8] (Fig 3). Peptides Hs03 and Hs04 induced similar thermal profiles in membranes among themselves and can be compared to the IAP O43312(33–62) [6] and the AMP Nattererin-1 [25] in our reference system [8]. Such biophysical analyses using model membranes indicate that the Hs IAPs prospected by the software Kamal are actual membrane disrupting agents, as anticipated, but present different qualities of membrane interaction.

### Hs IAPs are broad antimicrobial agents

The antimicrobial activities of the IAPs Hs01, 02, 03 and 04 were evaluated against a panel of human pathogenic microorganisms, comprising the yeast *C*. *albicans*, the filamentous fungi *T*. *rubrum*, the Gram-positive bacteria *S*. *aureus* and *S*. *epidermidis*, as well as the Gram-negative bacteria *P*. *aeruginosa* (Table 3). The frog skin peptides Asc-08 [26] and DS01 [27] were used as controls, along with reference commercial antifungal and antibiotic agents. All Hs IAPs were antimicrobial towards tested microorganisms with varying potencies (Table 3). IAPs Hs02 and Hs04 presented minimum inhibitory concentrations (MICs) comparable to reference AMPs (Table 3). Indeed, Hs02 was the most potent peptide against all evaluated microorganisms, including control AMPs. Moreover, Hs02 was the only molecule with significant inhibitory activity towards *P*. *aeruginosa*, indicating a wide spectrum of activity.

**Table 3:** Minimum inhibitory concentration towards microorganisms and hemolytic activity of Hs IAPs.

	Minimum inhibitory concentration (μM ± SD)					Hemolytic activity SC_50_*
	*C*. *albicans*	*T*. *rubrum*	*S*. *aureus*	*S*. *epidermidis*	*P*. *aeruginosa*	
Hs01	64.0 ± 0.0	NA	128.0 ± 0.0	64.0 ± 0.0	NA	> 128 μM
Hs02	1.67 ± 0.57	8.0 ± 0.0	1.0 ± 0.0	1.0 ± 0.0	1.67 ± 0.57	> 128 μM
Hs03	32.0 ± 0.0	256.0 ± 0.0	32.0 ± 0.0	21.33 ± 9.24	NA	> 128 μM
Hs04	8.0 ± 0.0	32.0 ± 0.0	4.0 ± 0.0	5.33 ± 2.31	NA	64 μM
Asc-8	10.67 ± 4.61	-	1.67 ± 0.57	4.0 ± 0.0	4.0 ± 0.0	32 μM
DS01	5.33 ± 2.31	128.0 ± 0.0	8.0 ± 0.0	4.0 ± 0.0	2.0 ± 0.0	> 128 μM
Fluconazol	1.63 ± 0.0		-	-	-	-
Amphotericin b	0.27 ± 0.0		-	-	-	-
Ampicilin	-	-	5.38 ± 0.0	43.10 ± 0.0	NA	-
Gentamicin	-	-	0.26 ± 0.0	0.26 ± 0.0	0.13 ± 0.0	-

* IAP concentration that at least 50% of red blood cells remain intact.

### Investigations on the cytotoxicity of Hs IAPs, with emphasis in the IAP Hs02

**Fig 4:**
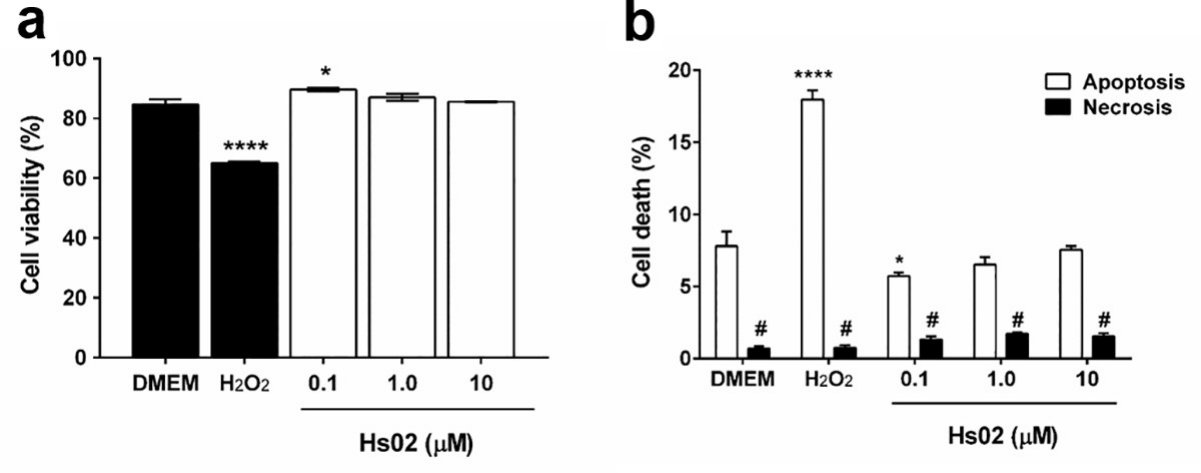
**a.** Cytotoxicity evaluation in the mouse BALB/cN macrophage (J774A.1) cell line after exposure for 24 h at 0.1, 1.0 and 10 μM concentrations. DMEM was used as a negative control. Cells were analyzed by flow cytometry (20,000 events/sample). **b**. Evaluation of the cell death mechanism mediated by Hs02 in J774A.1 cells after exposure for 24 h at 0.1, 1.0 and 10 μM concentrations using annexin-V FITC (apoptosis marker) and propidium iodide (PI, necrosis marker) staining. Cells were analyzed by flow cytometry (20,000 events/sample). The values are expressed as mean ± SEM. * *p*<0.05 and **** *p*<0.0001 versus DMEM untreated control group. # *p*<0.05 versus apoptosis staining for each respective group.

The hemolytic effect of Hs IAPs on human erythrocytes was evaluated. Judging by the peptide concentration that induces 50% hemolysis (Table 3, SC_50_), Hs IAPs are in general as hemolytic as the reference AMP DS01 [27], in consonance with results obtained for other IAPs from plant proteins [7]. The MTT reduction assay was conducted in primary macrophages to assess putative cytotoxic effects of Hs IAPs to this cell type (S1 Fig). Hs03 and 04 reduced the viability of peritoneal macrophages to 50% at approximately 10 μM, while Hs01 and 02 produced the same effect even at the lowest concentration evaluated (125 nM). To further investigate the putative cytotoxicity detected for Hs02, this peptide was incubated with mouse BALB/cN macrophages (J774A.1) cells at 0.1, 1 and 10 μM concentrations and analyzed by flow cytometry. Fig 4A shows that Hs02 did not cause significant (*p*<0.05) reduction in the viability of mouse BALB/cN macrophages (J774A.1) up to 10 μM concentration when compared with a DMEM untreated control, while the hydrogen peroxide (H_2_O_2_) cell death control decreased significantly (*p*<0.05) the cell viability. Additionally, analyses using annexin-V FITC (apoptosis marker) and propidium iodide (PI, necrosis marker) staining were performed to distinguish apoptotic cells from necrotic cells by flow cytometry in control and experimental groups [28,29] (Fig 4B). Cells treated with the peptide did not present significant apoptosis or necrosis in relation to the DMEM control group, confirming the cell viability assay.

### Hs02 modulates LPS-induced release of inflammatory mediators

**Fig 5:**
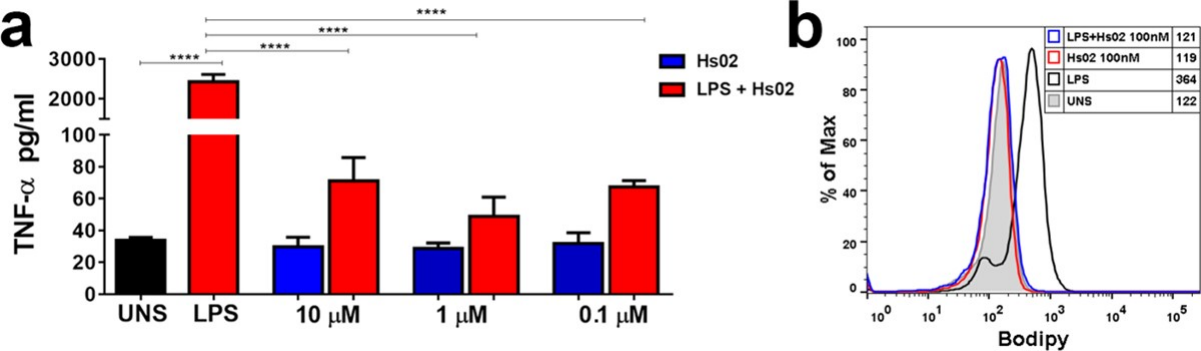
TNF-α secretion and lipid droplet biogenesis evaluation. **a.** Murine peritoneal macrophages from C57BL/6 mice were pre-treated or not with LPS (500 ng/ml) for 4h and then stimulated or not with Hs IAPs (0.1, 1.0 and 10 μM), incubated for 24h and TNF-α levels measured by ELISA **b.** In parallel, murine peritoneal macrophages from C57BL/6 mice were pre-treated or not with LPS (500 ng/ml) for 4h and then stimulated or not with Hs IAPs (0.1 μM), incubated for 24h and lipid droplets were quantitated by flow cytometry (B). The values are expressed as mean ±SEM. * *p*<0.05 and **** *p*<0.0001 versus DMEM control group.

Hs02 was evaluated in its capacity to elicit the release of inflammatory mediators in macrophages. No significant release of TNF-α, IL-1β, IL-17, or IL-6 was detected in THP-1 cells after stimulation with Hs02 at 1 μM concentration (S2 Fig). Additionally, Hs02 did not elicit the release of eicosanoids LTB_4_ or PGE_2_ in the same cell type at the same concentration. To investigate putative suppressive effects in the pro-inflammatory response induced by lipopolysaccharides in macrophages, cells were stimulated with 500 ng/mL LPS for 4h, and incubated with Hs02 at 0.1, 1 and 10 μM for 24h, followed by the quantification of TNF-α. As expected, LPS treatment of mouse macrophages resulted in a significant release of TNF-α (Fig 5A). Hs02 inhibited the release of TNF-α in LPS-primed cells even at the lowest peptide concentration evaluated, 0.1 μM (Fig 5A). Additionally, the ability of Hs02 to modulate cellular activation by investigation the lipid droplets biogenesis was analyzed. Within immune cells, LDs are considered as structural markers of inflammation [30] being able to store arachidonic acid, an essential component for the production of inflammatory mediators such as eicosanoids. Lipid droplet biogenesis of LPS-primed macrophages was quantified using the BODIPY dye assay coupled to flow cytometry. As expected, priming macrophages with LPS (Fig 5B, LPS) induced an increase of lipid droplet biogenesis compared to unstimulated macrophages (Fig 5B, UNS). Addition of Hs02 after 4h of the priming of macrophages with LPS at 0.1 μM concentration reduced LPS-induced lipid droplet biogenesis, reinforcing the anti-inflammatory activity of this peptide at this concentration (Fig 5B). DS01, our reference AMP, was unable to reduce LPS-induced release of TNF-α up to 10μM concentration, the highest concentration tested (S3 Fig).

### Hs02 structures as an α-helix in the presence of DPC-*d*_*38*_ micelles

The 3D structure of Hs02 was investigated by solution ^1^H NMR spectroscopy in DPC-*d*_*38*_ micelles. Except for residue K^1^, amino acid spin systems for the other 15 residues were identified and unequivocally assigned from the TOCSY and NOESY spectra (S4 Fig). The chemical shifts are reported in S1 Table and are deposited in the BMRB data bank with accession number 30509. The secondary structure of Hs02 was evaluated by calculating the chemical shift index (CSI) [31] of Hα backbone atoms, and it confirmed a propensity for helical structure spanning residues Ala^3^-Phe^14^ (S5 Fig). These results were also consistent with the connectivity obtained from the NOESY spectra. NOE panel (S5 Fig) and NOE restrains (Table 4) indicate medium-range NOEs, specially dαN(i, i+3), dαN(i, i+4) NOE, which are typical for helically folded peptides. An ensemble of micelle-bound structures was calculated using 291 inter-proton distance restraints and 24 dihedral angles. Statistical analyses of the constraints are described in Table 4.

**Table 4:** Structural statistics of the best 10 NMR structures of Hs02.

**NOE Restrains**	
Total number of distance Restraints	291
Number of intraresidue restraints	207
Number of sequential restraints (*i*, *i*+1)	40
Number of medium range restraints (*i*, *i*+j)_j = 2,3,4_	44
Dihedral angle	24
**RMSD (Å)**^**a**^ **–All residues**	
Backbone	0.51
Backbone and heavy atoms	1.27
**RMSD (Å)**^**a**^^,^^**b**^ **–Helical segment**	
Backbone	0.18
Backbone and heavy atoms	0.72
**Ramachandran plot analysis**^**a**^	
Residues in most favored regions	96.4%
Residues in allowed regions	2.9%
Residues in disallowed regions	0.7%

^a^Data from CCPNMR using 10 lowest energy structures

^b^from 3–14.

**Fig 6:**
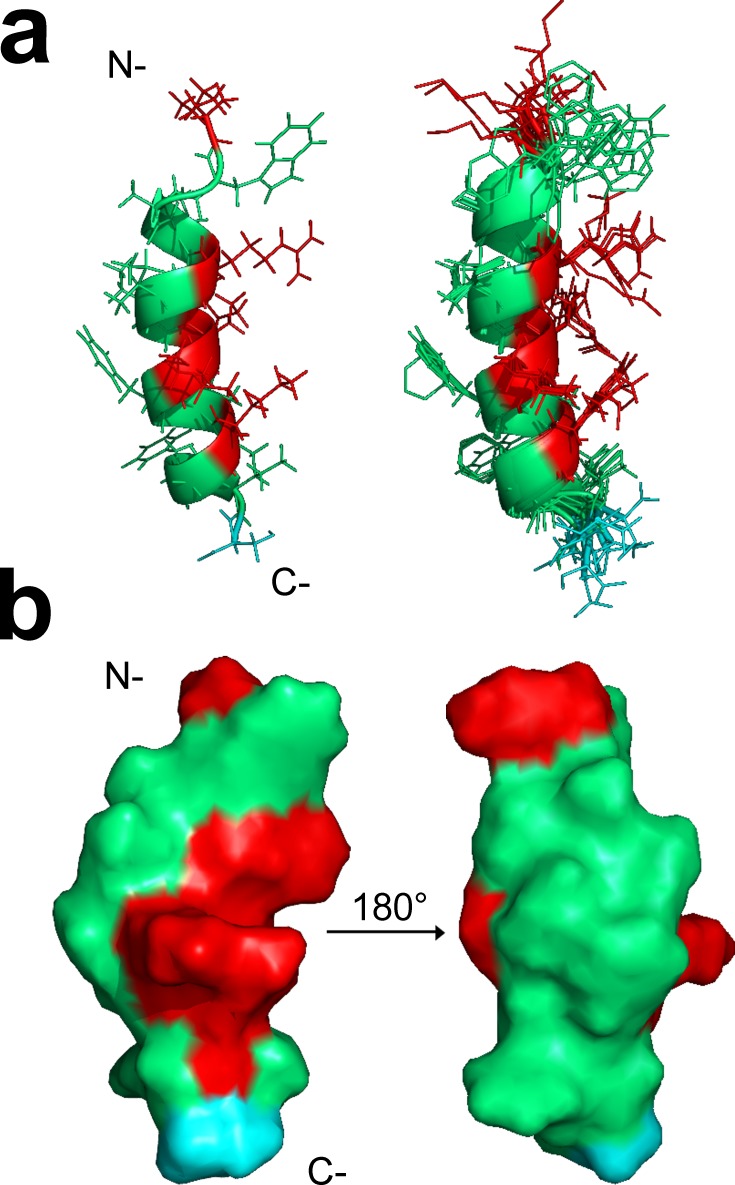
**a.** Ribbon representation of the lowest energy structure of Hs02 (left) and backbone alignment of the final 10 lowest-energy structures of Hs02 (right) in the presence of DPC-d38 micelles. **b.** Surface hydrophobic feature of Hs02 peptide. Side chain amino acid properties are showed in the following color code: red for the positively charged residues, cyan for the polar residues and green for the hydrophobic residues. The structure on the right side shows the molecule rotated 180° around the vertical axis (PDB: 6MBM).

Hs02 peptide has a well-defined structure in the presence of DPC-*d*_*38*_ micelles and adopts α-helix conformation ranging from residues Ala^3^-Phe^14^ (Fig 6A). It is interesting to note that its N-terminal region is disordered. As shown in Table 4, RMSD values drop significantly if the superposition is carried out over residues Ala^3^-Phe^14^, and indicate that the N-terminal has a random structure. An analysis of Ramachandran plot demonstrates that more than 96.4% of all residues are in the most favored regions of the diagram and 2.9% are in allowed regions, indicating a high stereochemical quality of NMR models. Only 0.7% of the residues are located in disallowed regions; however, these are located near the amino-terminus, which is structurally undefined. The pronounced amphiphilic nature of Hs02 is demonstrated in Fig 6B.

## Discussion

The present work describes the uncovering of Intragenic Antimicrobial Peptides (IAPs) encrypted in proteins encoded in the human genome, further exploring the antimicrobial activity, cytotoxicity, and putative immunomodulatory effects of representative molecules. While the release of antimicrobial fragments due to the partial hydrolysis of proteins from the human proteome has been described before [4,32,33], to our best knowledge, this is the first report of a large-scale *in silico* methodological-based enterprise to the uncovering of encrypted AMPs from human proteins. This work reinforces our previous claims that encrypted antimicrobial peptides are encountered in proteins from the most varied sources [6–8], and therefore not restricted to certain organisms or classes of molecules with involvement in the immune response.

The software Kamal was used to evaluate internal fragments of proteins encoded in the human genome that share similitude with α-helical amphiphilic AMPs [6–8]. The physicochemical properties under evaluation by Kamal and their reference ranges were determined in previous works [6,7] and are in constant evolution. In its current version, the software was programmed to identify amphiphilic segments of proteins using a set of amino acid rules, as an alternative to the calculation of the hydrophobic moment (μ_H_) [34] of internal sequences. Four Hs IAPs were chosen for chemical synthesis and biophysical investigations, mainly the determination of peptide structures in buffer and after the addition of a molar excess of model phospholipid LUVs, as well as the influence of peptides on the main phase transition of phospholipid vesicles. As expected from previous reports [6,7], the Hs IAPs varied in their secondary structure content in buffer, and in their extent of structuration as α-helical segments upon titration with DMPC and 2:1 DMPC:DMPG LUVs. By incorporating the Hs IAPs in the framework of membrane active peptides previously introduced by our group [8] it was possible to evaluate putative similarities in membrane-binding behavior of these molecules with previously acquired IAPs and AMPs using the same methodology. According to our model, the IAP Hs02 altered the main phase transition of model membranes (P´_β_→L_α_) similarly to Magainin-2a, as depicted in Fig 3. Both Magainin-2a and Hs02 do not affect significantly the main phase transition of DMPC vesicles, while they produce similar effects in the main phase transition of 2:1 DMPC:DMPG LUVs. The effect of 4 mol% Hs02 in membranes of the latter composition is consistent with a more superficial location [24], and further investigations should be carried out to evaluate the meaning and the extent to which Hs02 and Mag-2a are similar in mechanistic terms.

The antimicrobial activity of Hs IAPs towards common human pathogenic microorganisms was also evaluated. The MICs determined for these microorganisms were comparable to other IAPs obtained from plant proteins, likewise their hemolytic activities [7]. Unsurprisingly, the Hs IAPs presented wide antimicrobial spectra, being active against Gram-positive and -negative bacteria as well as yeasts, reinforcing our previous claims that these peptides are capable to hinder the growth of microorganisms despite their varied membrane structures [8]. Due to its pronounced activity and amplitude of antimicrobial spectrum, the IAP Hs02 was chosen for further evaluation. Although hemolysis was not pronounced for Hs02, which points to a high selectivity index, MTT of primary murine macrophages exposed to this molecule indicated significant toxicity. Potent amphiphilic α-helical AMPs are often cytotoxic [35], which prompted us to evaluate this subject further by flow cytometry using the J774A.1 macrophage cell line. Hs02 was tolerated up to 10μM concentration by this cell line, suggesting that putative fundamental compositional differences between primary murine macrophages and the J774A.1 macrophage cell line might make the former cell lineage more susceptible. MTT assays were also conducted in HaCat cells (S6 Fig), and statistically significant cytotoxicity for this peptide was observed only at 10μM concentration. No significant apoptosis or necrosis was induced by Hs02 up to 10μM concentration in J774A.1 in relation to DMEM control as shown by annexin and PI analyses. The traditional apoptosis pathway is a form of programmed and organised cell death that prevents triggering of local inflammatory process, while necrosis is a form of non-regulated cell death that causes cell lysis and exposition of its content [36]. In any case, Hs02 should be considered as a proof-of-concept, since human proteins might hold other encrypted IAPs with still possibly higher selectivity and potency towards microorganisms.

The anti-inflammatory effect of Hs02 in murine macrophages primed with LPS was also investigated. Lipopolysaccharide-neutralizing activity has been observed in several AMPs, and is now considered a common feature to this class of molecules [37]. The LPS-neutralizing ability of AMPs is often attributed to direct binding and dissolution of LPS aggregates, which reduces the TLR4 agonist capacity [37]. It is noteworthy that Hs02 displayed significant TNF-α suppressing activity in murine macrophages already primed for 4h with LPS, inhibiting the release of this cytokine at peptide concentrations as low as 0.1 μM. As demonstrated for other synthetic cationic peptides [38], Hs02 has thus not only prophylactic, but putative therapeutic potential as a potent anti-inflammatory molecule in sepsis models.

Once CD results showed that the Hs02 peptide adopts a helical conformation upon titration with model membranes, its structure was further investigated by solution NMR spectroscopy in the presence of DPC-*d*_*38*_ micelles. ^1^H NMR data showed that, in the presence of DPC micelles, Hs02 achieved a well-defined secondary structure characterized by the presence of an α-helical fold that spans throughout residues Ala^3^-Phe^14^, besides a prominent amphiphilic pattern, with a polar face enriched by positively charged residues (Arg^5^, Arg^8^, Lys^9^, Lys^12^). However, the percent helicity estimated by CD for Hs02 in DMPC LUVs is lower from that obtained for DPC-*d*_*38*_ micelles in the NMR solution structure. This might be due to the higher spacing of phospholipids headgroups in micelles in relation to lipid bilayers, and to the different peptide/lipid molar ratios used in CD and NMR experiments. According to the NMR data, the N-terminal region of Hs02 peptide exhibited a more flexible structure. Indeed, the Trp^2^ residue has a flexible side chain and, therefore, the position of its aromatic ring was not well defined, ranging from both the hydrophobic to the hydrophilic region of the molecule. The Trp sidechain is known to have an affinity for interfacial regions of lipid bilayers [39], which may increase its mobility.

The present work is based on the premise that proteins encoded by the human genome present amphiphilic α-helical structural segments, which, decrypted from their parent proteins and chemically synthesized as individual entities, can be used as antimicrobial and anti-inflammatory agents. Given that the membrane-permeabilizing and LPS-neutralizing activities commonly encountered in AMPs [37] are most likely due to their amphiphilic nature, we demonstrate that IAPs are actual alternatives to HDPs as pharmaceutical agents or in their many applications. Although the results presented herein should be viewed as a proof-of-concept, our findings suggest that the human genome holds an unexplored potential as a source of bioactive compounds with biotechnological potential in human health. Novel IAPs with more pronounced selectivity to microorganisms and anti-inflammatory potential are yet to be uncovered as novel tools for their recognition and testing are perfected or made available.

## Materials and methods

### Peptide filtering, selection and solid phase synthesis

Proteins were downloaded in .FASTA format from The UniProt database (www.uniprot.org) in October 2017, by searching for the keyword *Homo sapiens* under the term Organism (OS). The filtering of putative IAPs was performed using the software Kamal v 2.0 (http://www.cenargen.embrapa.br/kamal/) operating in mode 1. Proteins were scanned for putative encrypted IAPs ranging from 16 to 22 amino acid residues with the characteristic periodicity of α-helical amphiphilic segments, considering three possible polar angles, θ = 192°, 160° and 128°. Amino acid rules were created for the hydrophobic and hydrophilic faces based in a hydrophobicity scale [40]: amino acids Ala, Phe, Ile, Leu, Met, Val, and Trp were allowed in the hydrophobic face, while the hydrophilic face was less restrictive, allowing Ala, Asp, Glu, Gly, His, Lys, Met, Asn, Gln, Arg, Ser, Thr, and Tyr. Any putative segment containing either Pro or Cys was discarded. Additionally, only peptides with average hydrophobicity from -0.5 to 0.7 (TM scale [40]), net charge from +2 to +6, and aggregation tendency from -20 to +50 (Na4vSS parameter, Aggrescan algorithm [41]) were selected. These physicochemical parameters were chosen based on previous works by our group [6–8]. Four molecules were selected and chemically synthesized using Fmoc/t-butyl strategy [42]. The AMPs DS01 [27] and Asc-8 [26], were also synthesized and used as references. Peptide chain elongation was performed on Rink Amide resin, yielding C-terminal amidated peptides.

### Mass spectrometry analyses, HPLC purification and quantification

Synthetic peptides were analyzed by mass spectrometry to confirm peptide mass and amino acid sequence. Hs01-04 were individually mixed and co-crystallized with α-cyano-4-hydroxycinnamic acid matrix (Fluka) at 10 mg.mL^-1^ in a MALDI target plate. Experiments were carried out in an UltrafleXtreme MALDI-TOF/TOF (Bruker Daltonics). Peptides monoisotopic mass were obtained in reflector mode over a range of 700–3500 m/z with external calibration using Peptide Calibration Standard II (Bruker Daltonics). Peptide primary structures were inferred by means of manual interpretation of fragmentation spectra.

Reverse phase HPLC (RP-HPLC) of the synthetic peptides was performed in a LC-20A Prominence (Shimadzu Co.) using a Jupiter C_18_ 10 μm column (Phenomenex) at a flow rate of 10 mL.min^-1^. Ultrapure Milli-Q water and acetonitrile (J.T. Baker) added with 0.1% (v/v) trifluoroacetic acid (TFA) were used as solvent A and B, respectively. Fractions were manually collected and analyzed by mass spectrometry to confirm the elution time of each synthetic peptide.

Peptides containing Trp or Tyr residues were quantified using calculated molar absorption coefficients [43]. The remaining peptides were quantified using the UV absorbance of the peptide bond according to the literature [44].

### Differential scanning calorimetry (DSC) and circular dichroism (CD) assays

DMPC and 2:1 DMPC:DMPG (w/w) large unilamellar vesicles were prepared as described in the literature [6,7] and quantified using the ammonium ferrothiocyanate method [45]. Phospholipids were weighted, dissolved in chloroform, dried in a rotary evaporator, resuspended in 20 mM sodium phosphate–NaOH, 150 mM NaCl, pH 7.4, hand-shaken until the formation of a cloudy solution and passed through a 100 nm polycarbonate membrane using a mini-extruder. DSC analyses were also performed as previously described [7]. Briefly, thermograms were obtained using a VP-DSC (GE Healthcare) at a temperature range from 10 to 40 °C at a scanning rate of 0.5 °C/min. Peptides were added to fresh samples of 0.5 mM LUVs at a concentration of 20 μM (0.04 mol/mol peptide/phospholipids) at room temperature, immediately followed by DSC data acquisition. Each sample was subjected to various thermal scans until there were no distinguishable changes in the thermal profile of the main phase transition (P′_β_→L_α_) of phospholipids between individual scans. Data was concentration normalized, baseline subtracted (linear connect), and fit to a non two-state transition with two user determined peaks via the MicroCal Origin software v7.0. Re-scans for selected cases were acquired using fresh peptide and LUVs solutions to check the reproducibility of the data.

CD experiments were conducted on a Jasco-J815 spectropolarimeter (Jasco International Co.) as previously described [7]. Spectra were registered at room temperature from 200 to 260 nm as an average of 4 readings using a 0.1 cm path length cell, data pitch of 0.2 nm and a response time of 0.5 s. Data scans of buffer and 2 mM DMPC and 2:1 DMPC:DMPG LUVs solutions were acquired and subtracted from each peptide data. Peptides were scanned at a concentration of 40 μM in buffer and then 50 fold excess of DMPC and 2:1 DMPC:DMPG LUVs were added, resulting in a molar ratio of 0.02 peptide/phospholipid. The spectra were converted to mean residue ellipticity and readings at 222 nm ([θ]_222_) were used to estimate helix percentages [23].

### Evaluation of antimicrobial activity of IAPs

Protocols M7-A10 [46], M27-A3 [47] and M38-A2 [48] from Clinical & Laboratory Standards Institute (CLSI) were used to test the susceptibility of bacteria, yeasts and filamentous fungi to the Hs IAPs. Briefly, Hs IAPs ranging from 0.5 to 256 μM were tested against microorganisms in a final volume of 100μL. Tests were performed in polystyrene flat-bottom 96 wells microplates and each test consisted of three biological repetitions with 2 technical replicates each. The minimum inhibitory concentration (MIC) was defined as the concentration that no cells/hyphae were detected when visualized by optical microscopy. ATCC reference strains: *Candida albicans* 90028, *Staphylococcus aureus* 25923, *Pseudomonas aeruginosa* 27853, *Trichophyton rubrum* 28189, *Staphylococcus epidermidis* 12228.

### Cytotoxicity assay

The cytotoxicity of Hs IAPs was performed using human red blood cells in strict accordance with relevant guidelines and regulations (Ethical committee–UnB # 1.939.989 as described elsewhere [7]. Cytotoxic effect of IAPs was evaluated by MTT assay and flow cytometry analysis using murine peritoneal macrophages from C57BL/6 mice or a BALB/cN-derived macrophages (J774A.1) cell line. For cell viability assays using MTT, murine peritoneal macrophages from C57BL/6 mice were plated in triplicate wells in 96-well plates at a density of 1.0 × 10^5^ cells per well and stimulated or not with different concentrations of Hs IAPs (0.1, 1 and 10 μM) and incubated for 24h. After that, 20μl of a solution of 3-(4,5-Dimethyl-thiazol-2-yl)-2,5-diphenyl-tetrazolium bromide (MTT, Sigma) was added to each well (5 mg/ml of MTT in phosphate buffered saline). The plates were incubated at 37°C for 3 hours and then processed as previously described [49]. Cell death was analysed by annexin V-FITC/PI staining followed by flow cytometry analysis. Mouse BALB/cN macrophage (J774A.1) cell line was acquired from ATCC (number TIB-67) and cultured in Dulbecco’s Modified Eagle’s Medium (DMEM) (Life) supplemented with 10% heat inactivated foetal bovine serum (Life) and 1% antibiotic solution (100 IU/mL penicillin/100 μg/mL streptomycin, Life) at 37°C, 5% CO_2_, in a humidified atmosphere. J774A.1 cells were seeded into 96-well culture plates in DMEM culture medium for 2 h at 37°C and 5% CO_2_ in a humid atmosphere at a density of 1.0 × 10^5^ cells, and stimulated or not with different concentrations of Hs IAPs (0.1, 1 and 10 μM). The plates were incubated for 24 h, washed in PBS, and resuspended in 100 μL of binding buffer (10 mM HEPES/NaOH pH 7.4, 140 mM NaCl, ad 2.5 mM CaCl_2_). Cells were then treated with 2 μL of annexin-V FITC in Aqueous buffered solution containing BSA and ≤0.09% sodium azide (BD Pharmingen) and 2 μL of propidium iodide (PI, 50 μg/mL) for 15 min in the dark at room temperature. Next, 100 μL binding buffer was added and a total of 20,000 events were collected per sample in flow cytometer (BD LSRFortessa).

### TNF-α measurement by ELISA

The modulation of pro-inflammatory cytokine TNF-α secretion induced by IAPs was evaluated by ELISA. Murine peritoneal macrophages from C57BL/6 mice were pre-treated or not with LPS (500 ng/ml) for 4h and then stimulated or not with different concentrations of Hs IAPs (0.1, 1 and 10 μM) and incubated for 24 h. As a positive control, cells were stimulated only with LPS (500 ng/ml) for 24h. Supernatant TNF-α concentration was detected by ELISA with a R&D Systems (USA) kit. Microtiter plates were coated overnight at room temperature with capture antibody and blocked with Reagent Diluent for 1 hour. Serially diluted samples were added to the wells in triplicate and incubated overnight at 4˚C. After extensive washing, the cells were incubated with detection antibody and then Streptavidin-HRP. After washing, substrate solution was added and the plates were incubated for 15 minutes at room temperature. Plates were read after adding the stop solution at 450nm using SpectraMax M3spectrophotometer (Molecular Devices).

### Lipid droplets biogenesis

The ability of IAPs to activate cells was evaluated by analyzing lipid droplet biogenesis, an inflammatory cell activation marker. The lipid droplets were quantitated by flow cytometry. Murine peritoneal macrophages from C57BL/6 mice were pre-treated or not with LPS (500 ng/ml) for 4h and then stimulated or not with Hs IAPs (0.1 μM) and incubated for 24 h. After, cells were dissociated with trypsin (GIBCO), washed with PBS, incubated with the Bodipy (Sigma Aldrich, 50 nM) diluted in PBS for 30 minutes at 4°C in the dark. Cells were washed twice with PBS, resuspended in 500μL of PBS and stored at 4°C until reading by FACS Calibur using the FITC channel. FlowJo software was used to plot data and determine the median fluorescence intensity (MFI).

### ^1^H NMR spectroscopy and structures calculations

The lyophilized Hs02 peptide was dissolved in 600 μL PBS buffer at pH 7.0 and H_2_O/D_2_O (90:10, v/v) to a final concentration of 2 mM in 50 mM of DPC-d38. Additionally, 0.5% sodium-2,2,3,3-d4-3-trimethylsilylpropionate (TMSP-d4) was added as chemical shift reference internal standard. All ^1^H NMR experiments were performed on a Bruker Avance III HD 600 spectrometer operating at 14 T for ^1^H, at 25 ºC. The assignment of the peptide resonance peaks were carried out by two-dimensional (2D) experiments: total correlated spectroscopy (TOCSY) and nuclear Overhauser effect spectroscopy (NOESY). 2D spectra were acquired with 4096 complex points and 512 τ_1_ increments. The H_2_O signal was attenuated with excitation sculpting using 180 water-selective pulse. TOCSY spectra were obtained with a mixing time of 80 ms and NOESY spectra were recorded with a mixing time of 200 ms. The spectra were processed using NMRFX Processor software [50] and the contour maps were visualized using CCPNMR (version 2.4) software [51]. ^1^H chemical shifts were assigned according to standard procedures [52]. The NOEs were characterized based on the height of the cross peaks. The upper bounds for the NOE constraints were calibrated based in the r^-6^ distance dependence of the NOE, in three classes: strong (≤ 1.72 Å), medium (≤ 3.2 Å) and weak (≤ 8.0 Å) [53]. Structures were calculated using ARIA (version 2.3.1)[54] and CNS (version 1.2)[55] software. Several cycles of ARIA were performed using standard protocols and, after each cycle, rejected restraints, violations and assignments were analyzed. The ensemble of 10 lowest energy structures was chosen to represent the peptide solution 3D structure. NMR structures were deposited at PDB (www.rcsb.org) and were assigned with PDB code 6MBM.

### Statistical analysis

Results are reported as mean ± SD or mean ± SEM unless otherwise noted. Comparisons between groups were performed using analysis of variance (ANOVA) followed by Bonferroni’s multiple comparison test using GraphPad Prism version 6.0 (GraphPad Softwares, USA), at confidence interval of 95% (p< 0.05 was considered significant).

## Supporting information

S1 Table Chemical shifts ^1^H-NMR (ppm) to the HS02 peptide in micelle solution DPC-*d38* 50 mM, at 25°C and pH 7.0. (PDF)

S1 Fig MTT assays for the Hs IAPs using murine peritoneal macrophages from C57BL/6 mice. Murine peritoneal macrophages from C57BL/6 mice were plated in triplicate wells in 96-well plates and stimulated or not with different concentrations of Hs IAPs and incubated for 24h. 3-(4,5-Dimethyl-thiazol-2-yl)-2,5-diphenyl-tetrazolium bromide (MTT, Sigma) was added to each well at 10% (5 mg/ml MTT in phosphate buffered saline). The plates were incubated at 37°C for 3 hours with a) Hs01, b) Hs02, c) Hs03 and d) Hs04, and then processed as previously described [49].
(TIF)

S2 Fig THP-1 human monocytes were stimulated with Hs02 (1 μM), incubated for 24h and cytokines a) IL-1β, **b)** IL-6, **c)** IL-17, **d)** TNF-α levels were measured by ELISA. Additionally, eicosanoids **e)** LTB_4_ **f)** PGE_2_ levels were measured by EIA. The values are expressed as mean ± SEM.
(TIF)

S3 Fig TNF-α release in murine macrophages. Murine peritoneal macrophages from C57BL/6 mice were pre-treated or not with LPS (500 ng/ml) for 4h and then stimulated or not with the AMP DS01 (10, and 1 μM), incubated for 24h and TNF-α levels measured by ELISA. The values are expressed as mean ±SEM. * *p*<0.05 and **** *p*<0.0001 versus DMEM control group.
(TIF)

S4 Fig HN region of **A)** ^1^H TOCSY and **B)** ^1^H NOESY spectra obtained for 2 mM of Hs02 in the presence of 50 mM of DPC-*d38* at 25°C and pH 7.0.
(TIF)

S5 Fig a. Pattern of NOE connectivities involving sequential and medium proton distances of Hs02. The thickness of the bar indicates the intensities of NOEs. **b.** Hα CSI for Hs02.
(TIF)

S6 Fig MTT assays for the Hs IAPs using HaCat cells. HaCat cells were plated in triplicate wells in 96-well plates and stimulated or not with different concentrations of Hs IAPs (0.1, 1 and 10 μM) and incubated for 24h. 3-(4,5-Dimethyl-thiazol-2-yl)-2,5-diphenyl-tetrazolium bromide (MTT, Sigma) was added to each well at 10% (5 mg/ml MTT in phosphate buffered saline). The plates were incubated at 37°C for 3 hours with the assayed peptides and then processed as previously described [49].
(TIF)

## Abbreviations

AMPs: antimicrobial peptides
HDPs: Host defense peptides
IAPs: Intragenic Antimicrobial Peptides
