# Supplementary material for: Intragenic antimicrobial peptides (IAPs) from human proteins with potent antimicrobial and anti-inflammatory activity
Source: PLoS One. 2019 Aug 6;14(8):e0220656. doi: 10.1371/journal.pone.0220656 (PMC6684085; doi:10.1371/journal.pone.0220656)
Supplement: S1 Table — (PDF) [file pone.0220656.s001.pdf]

Table S1. Chemical shifts ^{1}H-NMR (ppm) to the HS02 peptide in micelle solution DPC-*d38* 50 mM, at 25 °C and pH 7.0.

**Residue**	δ_{NH}	$\delta_{H\alpha}$	$\delta_{H\beta}$	δ_{others}
Lys1	-	-	-	-
Trp 2	7.81	4.64	-	δ 7.31; ε 10.62; ζ 7.35 / 6.82; η 6.93
Ala 3	8.35	3.75	1.07	-
Val 4	7.92	3.41	1.97	γ 0.91 / 0.84
Arg 5	7.88	3.86	1.80	γ 1.61; δ 3.04
Ile 6	7.58	3.69	1.81	γ 1.35 / 0.74; δ 0.66
Ile 7	7.89	3.57	1.82	γ 1.14 / 0.81; δ 0.68
Arg 8	8.45	3.74	1.79	γ 1.46 / 1.64; δ 3.05
Lys 9	7.63	3.87	1.82	γ 1.40 / 1.27; δ 1.53; ε 2.80
Phe 10	8.08	4.24	3.12	γ 7.09
Ile 11	8.43	3.56	1.90	γ 1.24 / 0.83; δ 0.74
Lys 12	8.16	3.84	1.78	γ 1.43 / 1.30; δ 1.66; ε 2.81
Gly 13	7.82	3.73 / 3.94	-	-
Phe 14	7.84	4.23	2.91 / 2.74	δ 7.03
Ile 15	7.79	3.86	1.91	γ 1.19 / 0.83; δ 0.73
Ser 16	7.80	4.18	3.76	-
