# Supplementary figures and images for: Intragenic antimicrobial peptides (IAPs) from human proteins with potent antimicrobial and anti-inflammatory activity
Source: PLoS One. 2019 Aug 6;14(8):e0220656. doi: 10.1371/journal.pone.0220656 (PMC6684085; doi:10.1371/journal.pone.0220656)

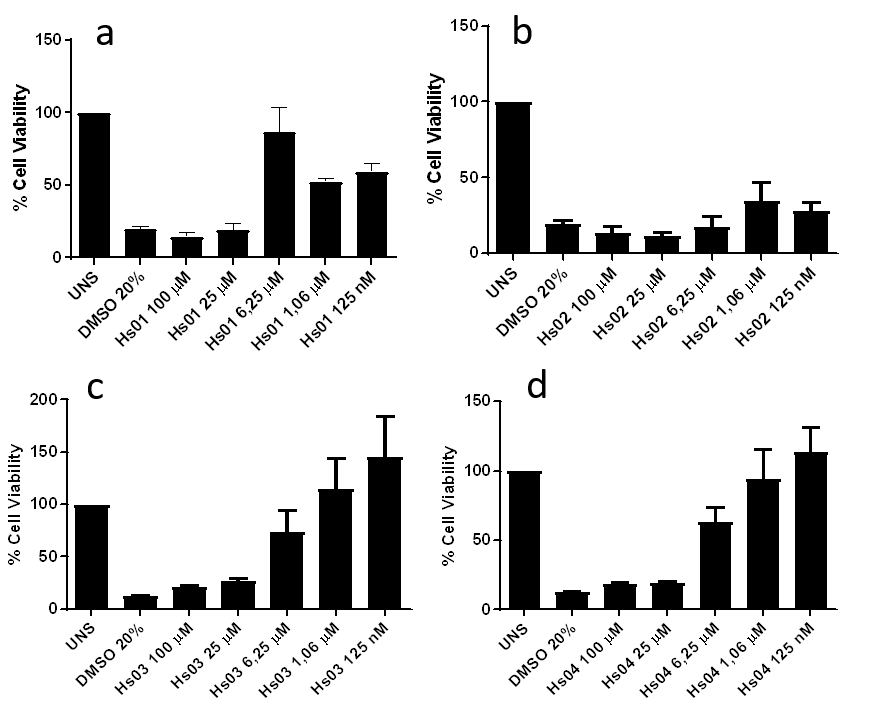

Supplement: S1 Fig — Murine peritoneal macrophages from C57BL/6 mice were plated in triplicate wells in 96-well plates and stimulated or not with different concentrations of Hs IAPs and incubated for 24h. 3-(4,5-Dimethyl-thiazol-2-yl)-2,5-diphenyl-tetrazolium bromide (MTT, Sigma) was added to each well at 10% (5 mg/ml MTT in phosphate buffered saline). The plates were incubated at 37°C for 3 hours with a) Hs01, b) Hs02, c) Hs03 and d) Hs04, and then processed as previously described [49]. (TIF) [file pone.0220656.s002.tif]

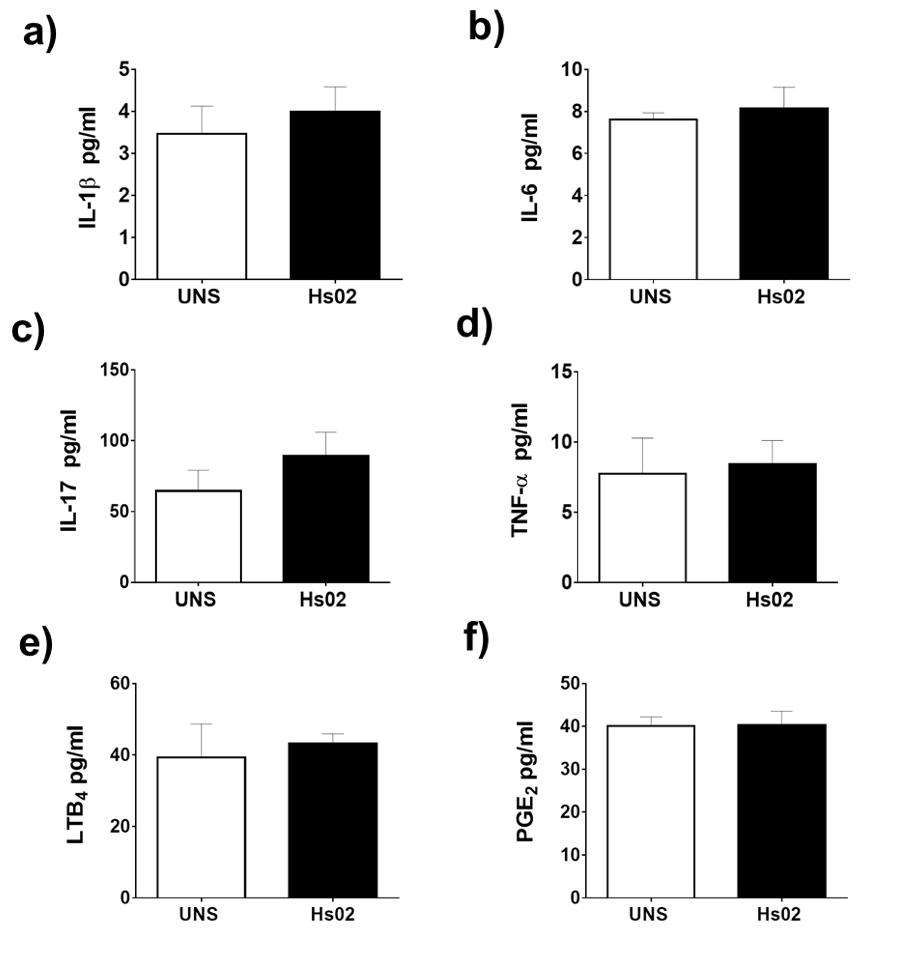

Supplement: S2 Fig — THP-1 human monocytes were stimulated with Hs02 (1 μM), incubated for 24h and cytokines a) IL-1β, b) IL-6, c) IL-17, d) TNF-α levels were measured by ELISA. Additionally, eicosanoids e) LTB4 f) PGE2 levels were measured by EIA. The values are expressed as mean ± SEM. (TIF) [file pone.0220656.s003.tif]

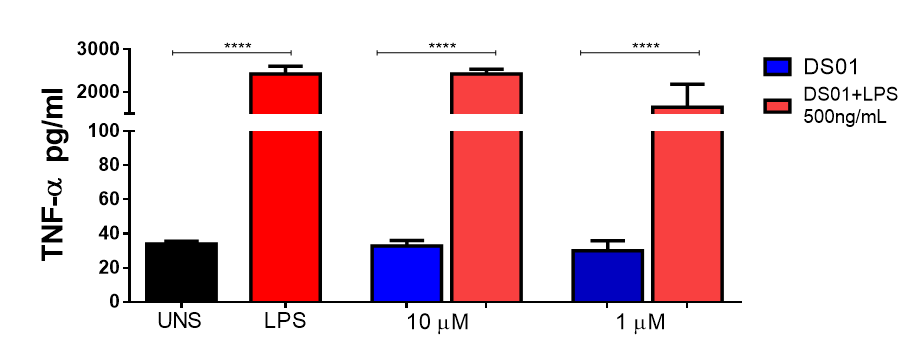

Supplement: S3 Fig — Murine peritoneal macrophages from C57BL/6 mice were pre-treated or not with LPS (500 ng/ml) for 4h and then stimulated or not with the AMP DS01 (10, and 1 μM), incubated for 24h and TNF-α levels measured by ELISA. The values are expressed as mean ±SEM. * p<0.05 and **** p<0.0001 versus DMEM control group. (TIF) [file pone.0220656.s004.tif]

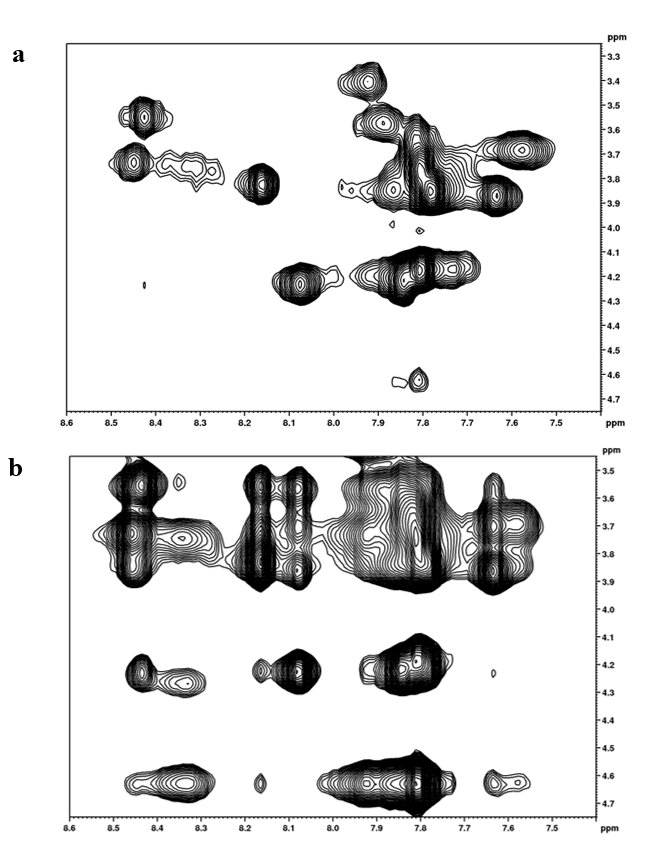

Supplement: S4 Fig — HN region of A) 1H TOCSY and B) 1H NOESY spectra obtained for 2 mM of Hs02 in the presence of 50 mM of DPC-d38 at 25°C and pH 7.0. (TIF) [file pone.0220656.s005.tif]

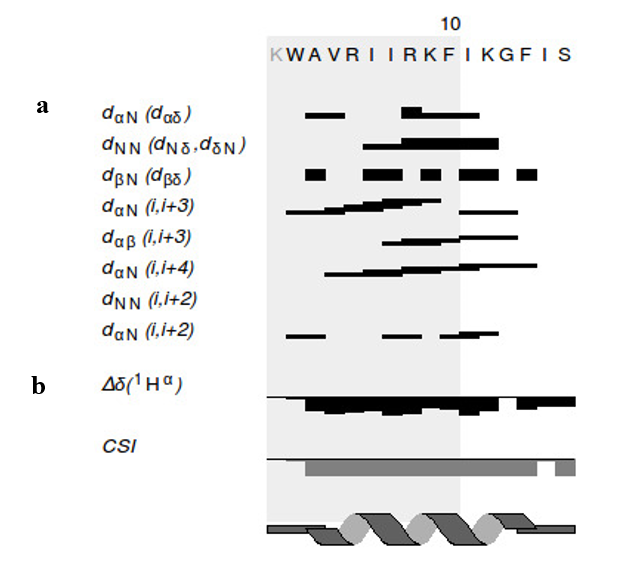

Supplement: S5 Fig — a. Pattern of NOE connectivities involving sequential and medium proton distances of Hs02. The thickness of the bar indicates the intensities of NOEs. b. Hα CSI for Hs02. (TIF) [file pone.0220656.s006.tif]

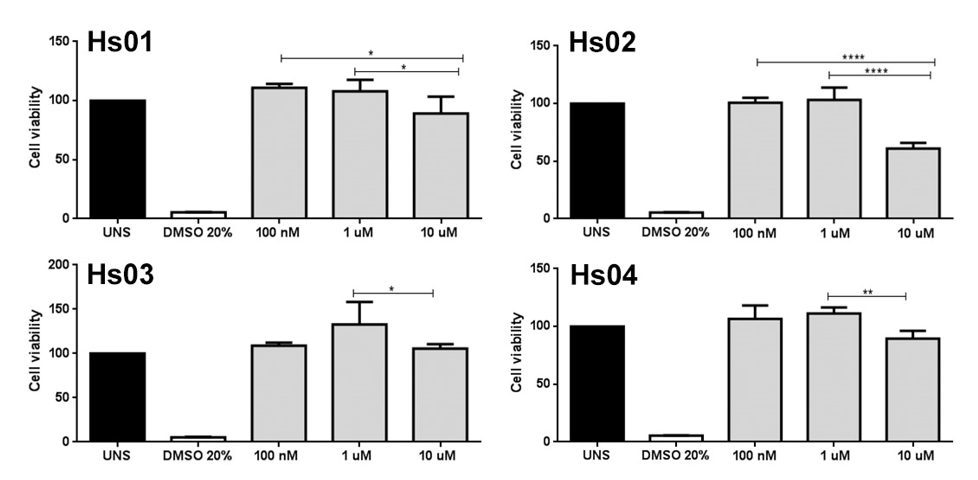

Supplement: S6 Fig — HaCat cells were plated in triplicate wells in 96-well plates and stimulated or not with different concentrations of Hs IAPs (0.1, 1 and 10 μM) and incubated for 24h. 3-(4,5-Dimethyl-thiazol-2-yl)-2,5-diphenyl-tetrazolium bromide (MTT, Sigma) was added to each well at 10% (5 mg/ml MTT in phosphate buffered saline). The plates were incubated at 37°C for 3 hours with the assayed peptides and then processed as previously described [49]. (TIF) [file pone.0220656.s007.tif]
